# Epidemiology of coinfection with tuberculosis and HIV in Japan, 2012–2020

**DOI:** 10.5365/wpsar.2022.13.1.896

**Published:** 2022-03-28

**Authors:** Lisa Kawatsu, Kazuhiro Uchimura, Noriyo Kaneko, Mayumi Imahashi

**Affiliations:** a Centre for Japan Pre-Entry Tuberculosis Screening Quality Assessment, Research Institute of Tuberculosis, Japan Anti-Tuberculosis Association, Tokyo, Japan.; b Department of Epidemiology and Clinical Research, Research Institute of Tuberculosis, Japan Anti-Tuberculosis Association, Tokyo, Japan.; c Department of Global and Community Health, Graduate School of Nursing, Nagoya City University, Nagoya, Japan.; d Department of Infectious Diseases and Immunology, Clinical Research Center, Nagoya Medical Center, Nagoya, Japan.

## Abstract

This report examines the characteristics and treatment outcomes of patients with tuberculosis (TB) who are coinfected with HIV in Japan. Active TB cases newly notified to the Japan Tuberculosis Surveillance system during 2012–2020 were analysed retrospectively, during which 379 HIV-positive TB cases were reported. The proportion of HIV-positive cases among those with known HIV status increased, from 1.9% (62/3328) in 2012 to 3.5% (31/877) in 2020. The proportion of those with unknown HIV testing status was consistently high, at approximately 60%, and the proportion of those who did not undergo HIV testing increased significantly, from 21.6% (4601/21 283) in 2012 to 33.7% (4292/12 739) in 2020. The proportion of foreign-born cases more than tripled, from 14.5% (9/62) in 2012 to 45.2% (14/31) in 2020. The TB treatment success rate was higher among HIV-negative than HIV-positive cases (72.7% [3796/5222] versus 60.3% [88/146]), and among Japan-born than foreign-born HIV-positive patients (65.6% [61/93] versus 50.9% [27/53]), owing largely to the high rate of foreign-born cases transferring to care outside Japan. The increasing proportion of HIV positivity among TB cases tested for HIV in this study requires ongoing monitoring, especially among foreign-born persons. However, because the number of reported cases was small, and there was low completeness of reporting of HIV testing data in the TB surveillance system, these results should be interpreted with caution. Encouraging more complete data collection by training public health nurses who complete TB case interviews and ensuring ongoing monitoring of patients with TB/HIV coinfection are recommended.

Tuberculosis (TB) continues to be a leading cause of death for people living with HIV or AIDS, and HIV remains the strongest known risk factor for progression to active TB disease for persons with latent TB infection. (*1*) In 2019, it was estimated that globally, 8.2% of all people with TB were living with HIV or AIDS. (*2*)

Japan has a middle-level, nearly low-level, burden of TB, with 12 739 cases newly notified in 2020, giving a notification rate of 10.1 per 100 000 population. (*3*) In the same year, 750 cases of HIV infection and 345 cases of AIDS were newly reported. (*4*) The number of new cases of TB, HIV and AIDS has continued to decline; however, there have been increases observed in the burden of these diseases among foreign-born persons. Because recent reports on the epidemiology of TB/HIV coinfection in Japan have been limited to regional analyses (*5*, *6*) or hospital-based studies, (*7*) we analysed national TB surveillance data to examine the characteristics and treatment outcomes of people with TB coinfected with HIV in Japan from 2012 to 2020.

## Methods

This was a cross-sectional study of active TB cases newly notified to the nationwide Japan Tuberculosis Surveillance (JTBS) system between 1 January 2012 and 31 December 2020.

### Japan Tuberculosis Surveillance system

The JTBS system was Japan’s first nationwide computerized TB surveillance system, introduced in 1987. As TB is a notifiable disease, new cases are notified to public health centres, which are responsible for collecting and entering case data into the system. The specific data items included in the JTBS system can be found elsewhere. (*3*) The JTBS data are summarized monthly and annually and are made publicly available online (https://jata-ekigaku.jp/english). Mechanisms to ensure data quality include an automatic verification programme and regular meetings attended by hospital and public health centre staff.

### HIV status

The information regarding a case’s HIV status upon diagnosis of TB is categorized as HIV-positive, HIV-negative, not tested for HIV and unknown HIV testing status. Entering these data is optional, and they are not cross-referenced with any other clinical database.

### Country of birth

Information regarding country of birth is reported as Japan-born, foreign-born or unknown. Foreign-born cases are defined as people, including Japanese citizens, who were born outside of Japan.

### Treatment outcomes

Prior to 2016, treatment outcomes were evaluated automatically using a computerized algorithm available only for pulmonary TB cases treated with a standard regimen. Since 2016, treatment outcomes have been entered directly by public health centres for all cases of active and latent TB. Therefore, this study examined treatment data from 2016 to 2020. Because the numbers of cases with treatment data available were quite small, statistical testing was not conducted to compare the proportions. One case with an unknown country of birth was excluded from the analysis.

### Data analysis and ethics

Trends in the epidemiological and clinical characteristics of TB cases notified in Japan were examined. HIV-positive TB cases, those not tested for HIV and those with unknown HIV testing status were compared. HIV-positive TB cases notified between 2016 and 2019 were analysed by country of birth, and their treatment outcomes were compared with those of HIV-negative TB cases. Trends were tested using the Cochran−Armitage test, and proportions were compared with tests for multiple comparisons, using the Hochberg correction to adjust the *P*-values. R version 3.6.3 (R Development Core Team, Vienna, Austria) was used for all statistical analyses.

## Results

### General trends, 2012–2020

**Fig. 1:**
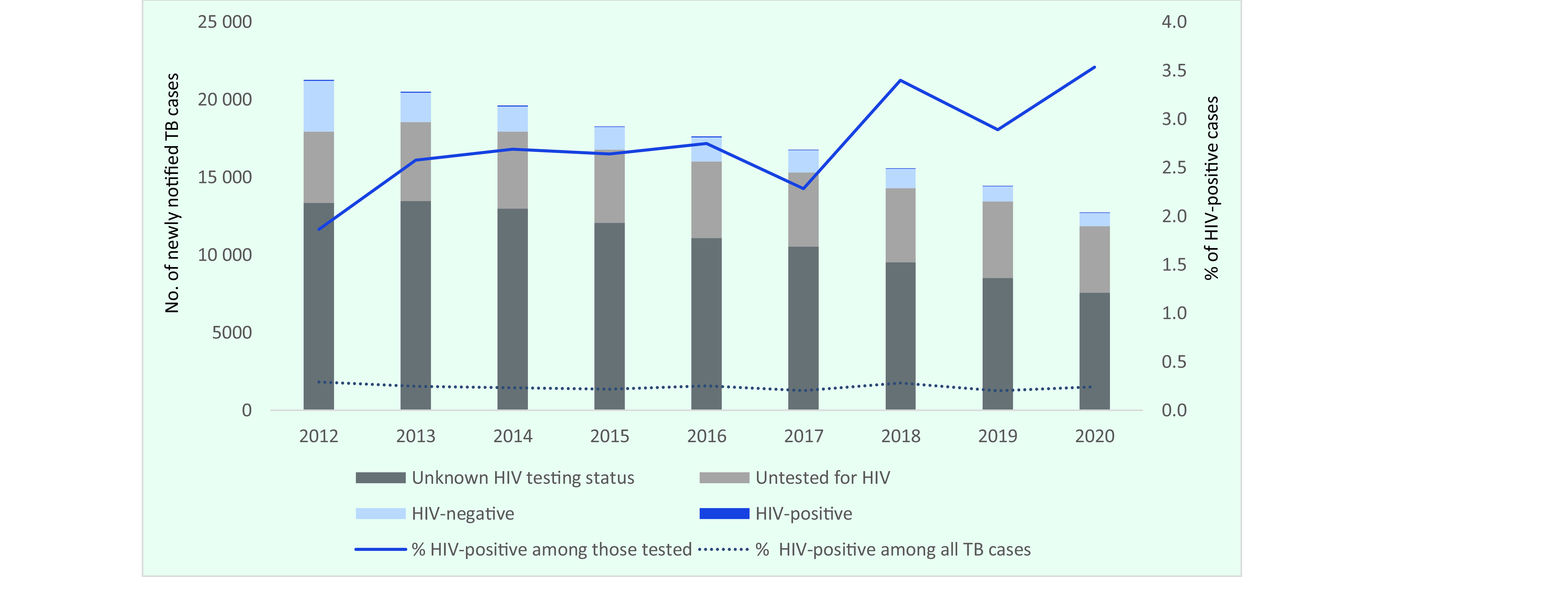
Number of newly notified TB cases by HIV status and proportion that were HIV-positive, by year, Japan, 2012–2020

**Fig. 2:**
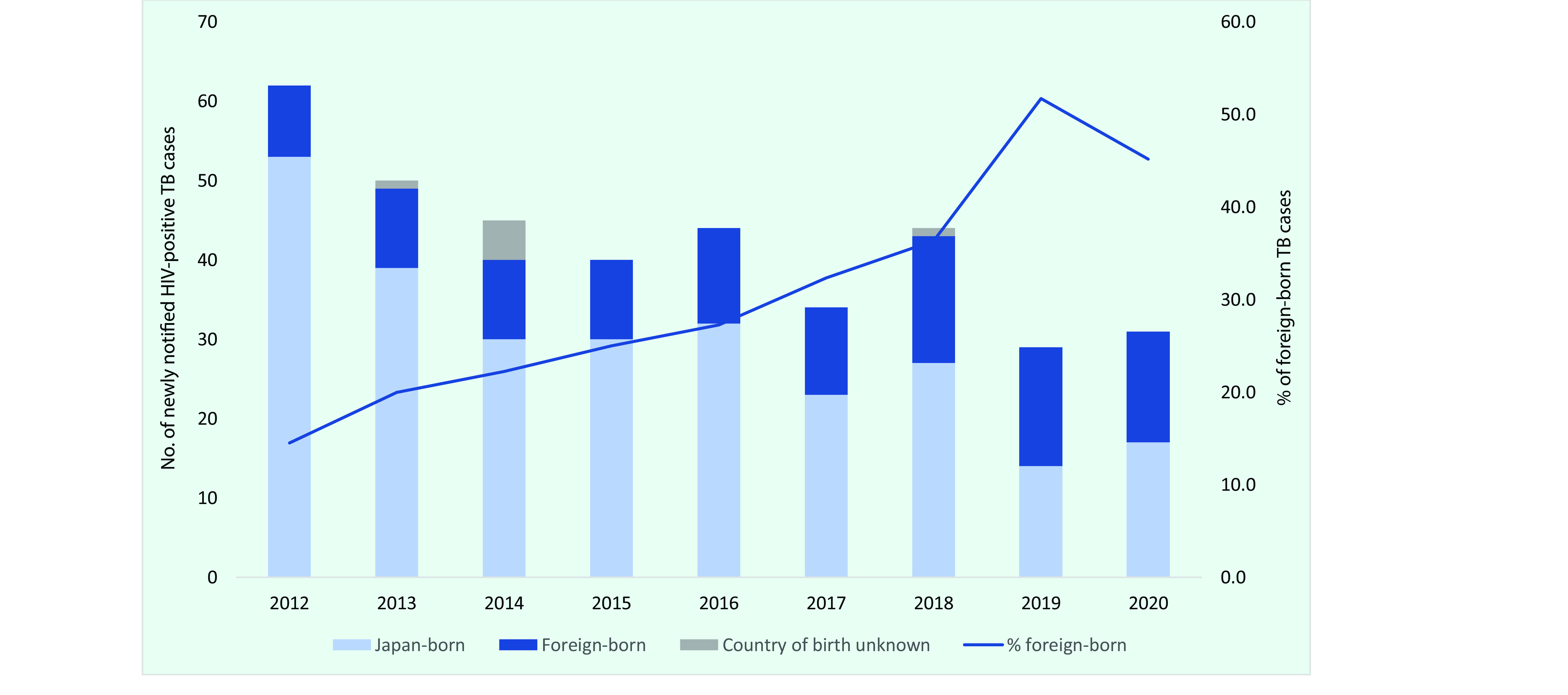
Number of newly notified TB cases by country of birth and proportion that were foreign-born, by year, Japan, 2012–2020

During the study period, a total of 156 876 TB cases were notified, of which 379 were categorized as HIV-positive, with the number of notifications steadily declining each year (**Fig. 1**). The proportion of HIV-positive cases among all TB cases was consistent at 0.2–0.3%; however, the proportion of HIV-positive cases among those tested for HIV increased significantly, from 1.9% (62/3328) in 2012 to 3.5% (31/877) in 2020 (*P* < 0.01). The proportion of those with unknown HIV testing status was consistently high, at 59.4% in 2020, whereas the proportion of those reported as not being tested for HIV increased significantly, from 21.6% (4601/21 283) in 2012 to 33.7% (4292/12 739) in 2020, although this decrease was not statistically significant (*P* < 0.01). The proportion of foreign-born cases among HIV-positive TB cases more than tripled, from 14.5% (9/62) in 2012 to 45.2% (14/31) in 2020 (**Fig. 2**).

[**insert** Figure 1]

[**insert** Figure 2]

### Proportions of cases by HIV status and selected characteristics

Among people with TB, 379 were categorized as HIV-positive, 14 339 were HIV-negative, 43 035 were not tested for HIV and 99 123 had unknown HIV testing status (**Table 1**). The proportion of those tested among the total TB cases was 9.4% (14 718/156 876). The proportion of HIV-positive cases among those tested was significantly higher in males (3.4% [313/9124]) versus 1.2% [66/5594]), highest among those aged 25–64 years old (4.8%; 290/5993) and highest for those whose country of birth was unknown (8.1%; 7/86), followed by those who were foreign-born (7.1%; 107/1505). Among occupational categories, the proportion of HIV-positive cases was highest among service industry workers (6.8%; 38/558), followed by temporary workers (5.1%; 34/666) (**Table 1**).

**Table 1:** Number and proportion of TB cases by selected characteristics and HIV status, Japan, 2012–2020

Characteristic	Notified TB cases							
	TOTAL	No. HIV-positive	No. HIV- negative	No. untested for HIV	No. HIV testing status unknown	% HIV-positive	% untested for HIV	% HIV testing status unknown
Total	156 876	379	14 339	43 035	99 123	2.6	74.5	63.2
**Sex**								
Male	94 677	313	8811	25 629	59 924	3.4	73.7	63.3
Female	62 199	66	5528	17 406	39 199	1.2	75.7	63.0
**Age group (years)**								
0–24	7059	17	836	2039	4167	2.0	70.5	59.0
25–64	46 534	290	5703	11 880	28 661	4.8	66.5	61.6
^3^65	103 283	72	7800	29 116	66 295	0.9	78.7	64.2
**Country of birth**								
Japan-born	139 521	265	12 862	39 215	87 179	2.0	74.9	62.5
Foreign-born	11 885	107	1398	3188	7192	7.1	67.9	60.5
Unknown	5470	7	79	632	4752	8.1	88.0	86.9
**Occupational category**								
Health-care worker	4526	15	478	1259	2774	3.0	71.9	61.3
Service industry worker	3916	38	520	1102	2256	6.8	66.4	57.6
Other full-time worker	22 524	98	2741	5884	13 801	3.5	67.5	61.3
Temporary worker	5256	34	632	1303	3287	5.1	66.2	62.5
Self-employed	6154	20	656	1714	3764	3.0	71.7	61.2
Unemployed								
Adults	103 310	152	8001	28 971	66 186	1.9	78.0	64.1
High school and university students	4360	11	636	1265	2448	1.7	66.2	56.1
Children and infants	527	1	45	225	256	2.2	83.0	48.6
Other or job unknown	6303	10	630	1312	4351	1.6	67.2	69.0

The proportion of those not tested for HIV was significantly higher in females than males (75.7%  [17 406/23 000] versus 73.7% [25 269/34 753]), highest in those aged (*3*)65 years old (78.7%; 29 116/ 36 988) and highest in those whose country of birth was unknown (88.0%; 632/718), followed by those who were born in Japan (74.9%; 39 215/52 342). Among occupational categories, the proportion of those not tested for HIV was highest among children and infants (i.e. excluding high school and university students) at 83.0% (225/271), followed by unemployed adults (78.0%; 28 971/37 124) (**Table 1**).

The proportion of those with unknown HIV testing status did not significantly differ by sex (63.3% [59 924/94 677] for males versus 63.0% [39 199/62 199] for females); however, it was highest among those aged (*3*)65 years old (64.2%; 66 295/103 283) and in those whose country of birth was unknown (86.9%; 4752/5470), followed by those who were born in Japan (62.5%; 87 179/139 521). Regarding occupational category, the proportion was highest among those in the category “Other or job unknown,” at 69.0% (4351/6303) (**Table 1**).

### Demographic characteristics of HIV-positive TB cases by country of birth

The majority of both the Japan-born and foreign-born HIV-positive TB cases were in the two younger age groups (0–24 years and 25–64 years). However, those aged 25–64 years accounted for 85.0% (91/107) of all foreign-born cases compared with 73.6% (195/265) of Japan-born cases. Males comprised 68.2% (73/107) and 88.3% (234/265) of foreign-born and Japan-born cases, respectively. For occupational categories for both Japan-born and foreign-born cases, the highest number of cases was among unemployed adults, followed by other full-time workers, service industry workers and temporary workers. These four occupational categories accounted for 86.8% (230/265) and 80.4% (86/107) of all Japan-born and foreign-born cases, respectively. Among foreign-born cases, high school and university students accounted for another 10.3% (11/107) of cases, but they were not represented in Japan-born cases (**Table 2**).

**Table 2:** Demographic characteristics of HIV-positive TB cases, by country of birth, Japan, 2012–2020 (*n* = 379)

Characteristic	Country of birth		
	Japan (*n* = 265)	Foreign country (*n* = 107)	Unknown (*n* = 7)
**Sex**			
Male	234	73	6
Female	31	34	1
**Age group (years)**			
0–24	3	14	0
25–64	195	91	4
^3^65	67	2	3
**Occupational category**			
Health-care worker	13	2	0
Service industry worker	26	12	0
Other full-time worker	67	29	2
Temporary worker	18	16	0
Self-employed	17	3	0
Unemployed			
Adults	119	29	4
High school and university students	0	11	0
Children and infants	0	1	0
Other or job unknown	5	4	1

### Treatment outcomes among TB cases by HIV status

The overall treatment success rate among HIV-positive TB cases was lower among foreign-born than Japan-born cases (50.9% [27/53] versus 65.6% [61/93]). Among Japan-born cases, the treatment success rate was lower in HIV-positive than HIV-negative cases (65.6% [61/93] versus 72.2% [3288/4557]), despite a larger proportion of those who were HIV-negative having died (8.6% [8/93] versus 19.0% [865/4557]). The lower treatment success rate among the HIV-positive cases was largely attributable to the higher proportion of those still on treatment (17.2% [16/93] versus 4.1% [185/4557] of HIV-negative cases still on treatment) (**Table 3**).

**Table 3:** Treatment outcomes for TB cases by country of birth and HIV status, Japan, 2016–2019

Treatment outcome	Country of birth			
	Japan, *n*(%)		Foreign country, *n*(%)	
	HIV-positive	HIV-negative	HIV-positive	HIV-negative
**Success**	**61 (65.6)**	**3288 (72.2)**	**27 (50.9)**	**508 (76.4)**
Cured	17 (18.3)	815 (17.9)	7 (13.2)	161 (24.2)
Treatment completed	44 (47.3)	2473 (54.3)	20 (37.7)	347 (52.2)
Died	8 (8.6)	865 (19.0)	3 (5.7)	14 (2.1)
Failure	0 (0.0)	4 (0.1)	0 (0.0)	1 (0.2)
Lost to follow-up	4 (4.3)	89 (2.0)	1 (1.9)	7 (1.1)
Transferred out	2 (2.2)	122 (2.7)	15 (28.3)	96 (14.4)
Treatment ongoing	16 (17.2)	185 (4.1)	6 (11.3)	37 (5.6)
Unknown	2 (2.2)	4 (0.1)	1 (1.9)	2 (0.3)
**Total**	**93 (100.0)**	**4557 (100.0)**	**53 (100.0)**	**665 (100.0)**

Among foreign-born cases, again, the treatment success rate was lower among HIV-positive than HIV-negative cases (50.9% [27/53] versus 76.4% [508/665]). This was largely attributable to higher proportions who were still undergoing treatment (11.3% [6/53] versus 5.6% [37/665]), as well as those who had transferred out (28.3% [15/53] versus 14.4% [96/665]) (**Table 3**).

## Discussion

Japan has historically had a low HIV prevalence, at less than 0.1 per 1000 population aged 15–49 years, (*8*) with a concentrated epidemic among men who have sex with men (MSM); (*9*) therefore, TB/HIV comorbidity has had less public health importance in Japan compared with other similarly industrialized countries. However, this study has shown an increasing proportion of HIV positivity among TB cases who were tested for HIV, which was 3.5% of those notified in 2020. This positivity rate is higher than in the United Kingdom of Great Britain and Northern Ireland (2.8% in 2018), but lower than in the United States of America (4.7% in 2019). (*2*)

The overall increase in HIV positivity in Japan may be due to the increase in the number of foreign-born persons with HIV who are in Japan: HIV positivity increased by 46%, from 91 cases in 2000 to 133 cases in 2019. (*4*) There have also been changes in the countries of birth of foreign-born persons with HIV in Japan between 2008 and 2013, with numbers decreasing from countries in the World Health Organization’s South-East Asia Region and increasing from countries in the Western Pacific Region, where the estimated number of people living with HIV has increased in recent years. (*8*, *10*) Issues associated with diagnosing HIV among foreign-born persons in Japan suggest that cases may be underreported. One study estimated that approximately 50% of HIV cases among foreign-born persons in Japan are currently diagnosed, (*11*) as opposed to approximately 80% among those born in Japan. (*9*) This estimate, along with delays in seeking HIV care among foreign-born cases (*8*, *10*) and the findings of this study, suggest that Japan could potentially be facing a large pool of foreign-born persons at risk of TB/HIV coinfection.

A low HIV testing rate (9.4%) was observed among TB cases in this study. Although HIV testing is recommended for all people with TB in Japan, (*12*) and questions about comorbidities are asked during the TB case interview, anecdotal evidence suggests that in practice, public health nurses are reluctant to counsel TB cases about HIV and offer testing. Because approximately 70% of Japan’s TB cases are aged (*3*)65 years, they are not perceived to be at risk of HIV infection or AIDS. Therefore, the question may seem unnecessary. Despite HIV testing being provided free of charge and anonymously at public health centres, low uptake has been reported in Japan (*13*, *14*) among younger populations and among MSM, who are considered to be more knowledgeable and conscious about the risk of HIV infection. (*15*) The number of HIV tests conducted at these centres has also gradually decreased, from about 146 000 tests in 2008 to 105 000 tests in 2019. (*4*) The proportion of adult males who have ever had an HIV test in Japan is around 10%, (*16*) much lower than in the United States (41.3%), (*17*) Canada (40.4%) (*18*) and England (32.4%). (*19*) Inconvenience, social stigma against homosexuality and discrimination against MSM and people living with HIV or AIDS are some reasons for the low uptake of testing, (*9*) and these may prevent the active promotion of HIV counselling and testing, even to those considered to be at high risk. It is necessary for public health centres to have staff who are adequately trained and have counselling skills to communicate with patients with TB about HIV and AIDS in multiple languages.

Treatment success differed by HIV status and country of birth. Treatment success was lower for Japan-born HIV-positive TB cases, at 65.6%, compared with Japan-born HIV-negative cases, at 72.2%, largely due to the high proportion of HIV-positive TB cases who were still undergoing treatment. Since treatment outcomes are entered into the JTBS system after 1 year of surveillance, it is possible that the final treatment outcomes of TB cases with HIV coinfection are not captured, especially as their treatment may have been prolonged due to complications.

Among foreign-born patients with TB, treatment success for HIV-positive patients, at 50.9%, was lower than that for HIV-negative cases, at 76.4%. Cases not in the category of treatment success include those still undergoing treatment and those who have transferred out of the system, for example, to return to their home country for treatment. Although there are various public subsidies for antiretroviral therapy in Japan, it is not completely free, and the process of applying for these subsidies can be complicated for people born outside of Japan. Thus the financial burden of antiretroviral therapy, coupled with psychological stress owing to difficulties in communication and cultural differences, have encouraged people who are HIV-positive, and also prompted physicians to persuade them, to return to their countries of birth to continue treatment. (*20*, *21*) To prevent disruption of treatment and poor treatment outcomes, social protection measures are urgently required to ensure that foreign-born TB patients receive appropriate care and treatment in Japan; additionally, cross-border referral mechanisms are also needed.

### Limitations

Our study had several limitations. First, entering whether a person is living with HIV into the JTBS system is optional. Therefore, public health nurses who interview patients with TB are not obligated to ask for that information. Second, when HIV status is recorded, it is self-reported and not verified or linked to other clinical databases. Therefore, it is possible that the HIV positivity rate reported among TB cases is an underestimation of the true occurrence in Japan. Also, because there are no data in Japan about the uptake of HIV testing stratified by sociodemographic characteristics, it is difficult to assess whether the increase in HIV positivity among those tested was due to a real increase in the number of HIV-positive TB cases or if HIV testing was more focused on high-risk groups. (*22*)

## Conclusions

The increasing proportion of HIV positivity among TB patients tested for HIV shown in this study requires ongoing monitoring, especially among foreign-born persons. However, as the number of reported cases was small, and the data on HIV testing reported in the JTB system were not complete, these results need to be interpreted with caution. Encouraging more complete data collection by training the public health nurses who complete TB case interviews, as well as ensuring there is ongoing monitoring of patients with TB/HIV coinfection is recommended.
